# Depressive symptoms are associated with achieving remission in PsA patients – an analysis from the RABBIT-SpA register

**DOI:** 10.1186/s41927-026-00707-y

**Published:** 2026-10-14

**Authors:** Andreas Reich, Anja Weiß, Lisa Lindner, Martin Feuchtenberger, Georg Dahmen, Hanns-Martin Lorenz, Xenofon Baraliakos, Anne C. Regierer

**Affiliations:** 1 German Rheumatology Research Center (DRFZ Berlin), Epidemiology and Health Services Research, Charitéplatz 1, 10117 Berlin, Germany; 2 Charité – Universitätsmedizin Berlin, Berlin, Germany; 3 MVZ am InnKlinikum, Burghausen, Germany; 4 Medizinische Klinik und Poliklinik II, University Hospital, Wuerzburg, Germany; 5 Rheumatologist, Hamburg, Germany; 6 Division of Rheumatology, Department of Internal Medicine V, Heidelberg University Hospital, Heidelberg, Germany; 7 Ruhr University Bochum, Bochum, Germany; 8 Rheumazentrum Ruhrgebiet, Herne, Germany

**Keywords:** Depression, Sex factors, Treatment outcome, Arthritis, psoriatic, Biological products, Antirheumatic agents, bDMARD, tsDMARD, Observational study

## Abstract

**Background:**

Depressive symptoms are common in psoriatic arthritis (PsA) and may influence treatment response. This study examined their association with achieving remission after starting a new systemic antirheumatic therapy, and tested whether this association differs by sex.

**Methods:**

Data from PsA patients in the ongoing prospective longitudinal observational cohort study RABBIT-SpA were analyzed. Depressive symptoms were assessed with the WHO-5 Well-Being Index (none/mild vs. moderate/severe). Outcomes included minimal disease activity (MDA) and Disease Activity in Psoriatic Arthritis (DAPSA) remission at 3 and 6 months. Logistic regression assessed the association between baseline well-being and remission, adjusted for confounders identified using directed acyclic graphs. A sex × well-being interaction term was included in each model to test whether this association differs by sex. Multiple imputation handled missing data.

**Results:**

Moderate/severe depressive symptoms were present in 29% of 1,888 patients (34% of women, 23% of men). In adjusted analyses, higher baseline well-being was associated with remission (OR per 10-point increase: 1.06–1.14 across outcomes and timepoints, reaching significance for MDA at 6 months and DAPSA remission at both timepoints). Interaction testing showed no evidence that this association differed by sex; all four 95% confidence intervals included 1 (*p* ≥ 0.29). Both sexes with moderate/severe symptoms achieved MDA and DAPSA remission less frequently than those with no/mild symptoms. Although disease activity improved in both groups over time, it remained higher in patients with moderate/severe symptoms.

**Conclusions:**

Depressive symptoms were associated with higher disease activity and less frequent remission in PsA, with no evidence that this effect differed by sex. Routine screening for depressive symptoms should be considered in PsA management to identify patients at risk of suboptimal treatment response.

**Clinical trial number:**

Not applicable.

**Supplementary Information:**

The online version contains supplementary material available at https://doi.org/10.1186/s41927-026-00707-y.

## Background

Psoriatic arthritis (PsA) is a chronic inflammatory disease affecting up to 30% of psoriasis (PsO) patients. It is heterogeneous in its clinical manifestation and can present with peripheral arthritis, axial involvement, enthesitis, dactylitis, and nail involvement in addition to PsO [1].

PsA is associated with a markedly increased number of comorbidities, compared to control populations, particularly cardiovascular diseases, which in turn are associated with reduced quality of life and function and reduced life expectancy [2]. Mental health comorbidities like depression are frequent. A recent systematic review found a pooled estimate of prevalence of depression of 5% to 51%, using different instruments and cut-off values [3]. The prevalence of ‘at least mild’ depression was 20%, that of ‘at least moderate’ depression 14%. The prevalence was higher when screening instruments, like the WHO-5 Well-Being Index (WHO-5), were used vs. using documented or self-reported comorbidities [3]. In an earlier analysis from our register, 29% of PsA patients showed moderate/severe depressive symptoms, measured with the WHO-5 [4]. There is evidence that depressive symptoms have an independent, negative effect on response to antirheumatic therapy in patients with rheumatoid arthritis (RA) [5–7], axial spondyloarthritis (axSpA) [8, 9] and PsA [5, 6, 10]. While there are known sex differences in phenotype, treatment, comorbidities and response in PsA, it is not known whether the association between depressive symptoms and PsA outcome is sex-specific [11].

The primary objective of this analysis was to examine the association between baseline well-being and achievement of MDA or DAPSA remission at 3 and 6 months in patients with PsA.

Secondary objectives were (a) to test whether this association differs by sex, (b) to examine the proportions of patients achieving MDA or DAPSA remission by depressive symptom category, (c) to analyze the components of MDA and DAPSA over time (baseline, 3, and 6 months), and (d) to describe the proportion of patients achieving sustained MDA or DAPSA remission (remission at both 3 and 6 months).

## Methods

### Data source

The RABBIT-SpA register is a longitudinal, observational cohort of patients diagnosed with axSpA or PsA. The main objective of RABBIT-SpA is to examine the long-term effectiveness and safety of biological and targeted synthetic disease-modifying anti-rheumatic drugs (b/tsDMARDs) in real-world patients. Participants are recruited from rheumatological practices and clinics throughout Germany at the start of a new systemic treatment. The control group consists of individuals starting a new treatment with conventional synthetic DMARDs (csDMARDs) or non-steroidal anti-inflammatory drugs (NSAIDs).

Data collection involves both physician and patient case report forms (CRFs), which are completed at recruitment, at 3 and 6 months post-baseline, and subsequently every 6 months, with a follow-up period of up to 10 years per participant. Additional information regarding the design of the register and methodology can be found in previous publications [4, 12, 13].

### Patient and public involvement

The German Rheumatology Research Center Berlin (DRFZ) is in close collaboration with the Deutsche Rheuma-Liga, a German patient organization for individuals affected by rheumatic and musculoskeletal disorders. A patient research partner representative from the Deutsche Rheuma-Liga is included on both the DRFZ’s scientific advisory board and our register’s scientific advisory board.

### Patient recruitment

Patients were enrolled by the individual study centres and all patients provided informed consent in writing. All PsA patients enrolled since the initiation of our register in May 2017 until database closure on September 1st 2024 are included in this analysis, irrespective of their assignment to the b/tsDMARD or control group. Patients were included if they had a baseline physician’s CRF and enough follow-up time to have received a six month follow up. For this analysis, baseline refers to the start of observation in the register.

### Variables

We collected sociodemographic, lifestyle, clinical, treatment, and patient-reported outcomes (PROs).

#### Sociodemographic and lifestyle factors

Age (years) was recorded as a continuous variable. Body Mass Index (BMI) was calculated from patients’ height and weight and categorized as obesity (BMI ≥ 30 kg/m²) (yes/no). Smoking was captured as current smoking (yes/no). Education was categorized according to the International Standard Classification of Education (ISCED), with participants classified as having ISCED level 2 (up to 10 years of schooling) or ISCED level 3 (> 10 years of schooling) [14]. Employment status was coded as currently employed (yes/no). Engagement in sports was obtained from the patient CRF and dichotomized as sports, ≥ 1 h/week (yes/no). Information on sex was obtained from the physician questionnaire and is assumed to represent biological sex rather than gender. The variable included the categories ’male’, ’female’, and ’other’. Since none of the patients in this analysis were recorded as ’other’, the analyses are restricted to patients categorized as male or female. Throughout the manuscript, we therefore use the terms ’men’ and ’women’ to denote biological sex. No information on patients’ gender identity was available

#### Clinical characteristics

Patients reported the dates of first symptoms and first diagnosis for joint manifestations. From these, we calculated symptom duration and duration since diagnosis

Fulfilment of the Classification criteria for psoriatic arthritis (CASPAR) [15] was recorded as ‘CASPAR criteria satisfied’ (yes/no). Nail psoriasis was recorded (yes/no); current skin involvement was recorded as body surface area affected (BSA) (%). Enthesitis was documented as current enthesitis (yes/no) and severity was assessed using the Spondyloarthritis Research Consortium of Canada Enthesitis Index (SPARCC 0–16) [16].

Joint counts included tender joint count (TJC, 0–68) and swollen joint count (SJC, 0–66). For descriptive purposes, we additionally report the presence of any tender or swollen joints (≥ 1) and elevated joint counts (≥ 5). C-reactive protein (CRP) was measured in mg/L and analyzed as log-transformed CRP as well as CRP ≥ 5 mg/L.

Comorbidity data were obtained from the physician’s CRF, which included a checklist of relevant conditions such as cardiovascular diseases, renal diseases, malignancies, and depression and the option to add further comorbidities in a text field. Overall comorbidity burden was summarized as comorbidities, three or more (yes/no).

Physician global disease activity (PhGA) was assessed on a 0–10 numeric rating scale (NRS), with separate assessments for skin (PhGA Skin) and musculoskeletal manifestations (PhGA MSK) [17].

#### Patient-reported outcomes

Patient global disease activity (PtGA) was assessed on an NRS 0–10, with separate assessments for skin (PtGA Skin) and musculoskeletal symptoms (PtGA MSK), reflecting the demonstrated utility of assessing global disease activity separately for the skin and joint domains [18]. In the DAPSA and MDA definitions, PtGA and PtGA MSK correspond to the standard patient global assessment and pain items of the DAPSA patient questionnaire, respectively. Patients also rated their pain and fatigue on an NRS 0–10, using the first two items of the Psoriatic Arthritis Impact of Disease (PsAID) questionnaire [19]; the overall PsAID score (0–10, based on all 12 items, higher scores indicating greater impact) was additionally used to assess overall disease impact. Physical function was measured with the Health Assessment Questionnaire (HAQ, 0–3; higher scores worse) [20]. Skin-related quality of life was captured by the Dermatology Life Quality Index (DLQI, 0–30; higher scores worse) [21].

#### Treatment

Prior exposure to advanced therapies was coded as b/tsDMARD naïve (yes/no). The treatment at inclusion was categorized as inclusion therapy: NSAID, csDMARD, bDMARD, or tsDMARD. Current use of NSAIDs, csDMARDs, and glucocorticoids was recorded (yes/no).

#### Composite disease activity measures and outcomes

The DAPSA [22] score was calculated as the sum of TJC, SJC, PtGA, PtGA MSK, and CRP (converted to mg/dL). MDA [23] is defined as at least 5 of the following 7 criteria being fulfilled: TJC ≤ 1, SJC ≤ 1, psoriasis activity and severity index (PASI) ≤ 1 or PsO affecting BSA < 3%, PtGA MSK (VAS) ≤ 15 mm, PtGA VAS ≤ 20 mm, Health Assessment Questionnaire-Disability Index (HAQ-DI) ≤ 0.5, tender entheseal points ≤ 1. In our register, both PtGA MSK and PtGA are measured on an NRS 0–10, so we used < 2 as a cut-off for PtGA MSK and ≤ 2 for PtGA when defining MDA. We also used BSA as we do not have PASI data.

#### Depressive symptoms

Depressive symptoms were assessed with the WHO-5. While not a diagnostic tool, it is well-established and recognized as an instrument to screen for depression, in research as well as in clinical practice [24]. It consists of the following 5 items, measured on a scale from 0 (never) to 5 (all of the time): 1: ’I have felt cheerful and in good spirits’; 2: ’I have felt calm and relaxed’; 3: ’I have felt active and vigorous’; 4: ’I woke up feeling fresh and rested’; 5: ’My daily life has been filled with things that interest me’. The individual item scores are summed and then multiplied by 4 to yield a WHO-5 percentage score ranging from 0 to 100 with higher values indicating higher well-being. Based on established thresholds [25–27], patients were categorized into two groups: those with a score below 29 were classified as having moderate/severe depressive symptoms, those with a score of 29 or higher as having no/mild depressive symptoms. We use ‘well-being’ when referring to the continuous WHO-5 score (used as the exposure in the regression models) and ‘depressive symptoms’ when referring to the categorical classification (none/mild vs. moderate/severe) derived from it. Since patients in our register complete the WHO-5 annually, no additional assessments were available within the study’s analysis period

#### Remission

Remission was defined as achievement of MDA or DAPSA remission (DAPSA < 4) [28]. Sustained remission was defined as MDA/DAPSA remission after both 3 and 6 months, and is reported descriptively; it was not analyzed as a separate regression outcome in this study. All baseline variables were collected simultaneously within a two-week window before or after treatment initiation to accurately capture baseline disease activity before the new treatment could influence it.

### Statistical analysis

#### Missing data handling

Missing data were addressed using multiple imputation with chained equations (MICE), assuming data were missing at random (MAR). The imputation model included all analysis variables plus auxiliary variables (comorbid depression and fibromyalgia as documented by the rheumatologist, PhGA, DLQI, employment status, dactylitis, and CASPAR classification criteria) to make the MAR assumption more plausible. Repeated measures at baseline, 3, and 6 months (e.g., HAQ, joint counts, CRP) were included as separate variables within a single wide-format imputation model, following standard practice for imputing a modest number of repeated-measures timepoints [29]. No custom predictor-matrix restrictions were applied, so each timepoint’s values could inform imputation of missing values at other timepoints.

Predictive mean matching (PMM) was used for all variables, including binary and categorical variables, rather than variable-type-specific parametric imputation methods (e.g., logistic or polytomous regression) [29].

Baseline WHO-5 scores, missing for 251 of 2,139 patients, were imputed using the same procedure as other analysis variables. Imputing the exposure, rather than restricting the analysis to patients with observed WHO-5, retains statistical power and avoids the selection bias that could arise if patients with missing WHO-5 differ systematically from those with observed WHO-5 (see Additional file 1: Table S1 and our discussion of selection bias in the Strengths and limitations section). We generated 50 imputed datasets with 20 iterations each, exceeding the recommended minimum number of imputations based on the percentage of incomplete cases in the analysis with the highest missingness [30]. Analyses were performed separately on each imputed dataset and results were pooled using Rubin’s rules. The fraction of missing information was assessed for all model coefficients. Variable-level missingness and detailed imputation diagnostics are provided in Table 1 and Additional file 1: Table S3. All imputation procedures were performed using the mice package in R [31].

#### Directed acyclic graph

We used a directed acyclic graph (DAG) to guide confounder selection and clarify assumptions about the causal structure underlying the relationship between depressive symptoms, remission, and other patient characteristics. The initial DAG was constructed based on clinical expertise, relevant literature, and data availability, and included variables from the Outcome Measures in Rheumatology Core Domain Set for psoriatic arthritis [32]. However, we decided to omit fatigue, as it is a core symptom of our exposure, depressive symptoms, and including it could introduce bias in our analysis. We constructed separate DAGs for our two outcomes, MDA and DAPSA remission. Our regression models estimate the direct effect of baseline well-being on remission, that is, the effect not mediated through the choice of inclusion therapy (b/tsDMARD vs. standard therapy). Accordingly, our adjustment sets exclude inclusion therapy, which we model as a mediator rather than a confounder [Additional file 1: Figures S5, S6].

#### Descriptive statistics

Baseline characteristics (Table 1) are presented stratified by depressive symptom category (WHO-5 < 29 vs. ≥29) and sex, using the observed data. For continuous variables we report mean and standard deviation (SD) for normally distributed variables and median (Q1-Q3) for skewed distributions. For binary and categorical variables, we report counts and proportions. The percentage of available data (non-missing observations) is reported for each variable. The time course of mean component values (Fig. 3) is presented descriptively, as a complement to the primary regression analyses, which analyze the association between baseline well-being and remission using MDA and DAPSA remission as outcomes. The proportion of patients achieving sustained MDA or DAPSA remission (remission at both 3 and 6 months; Fig. 2, Additional file 1: Figure S2) is also presented descriptively.

#### Logistic regression

In the regression models analyzing the association between well-being and achievement of remission, baseline well-being was modelled as a continuous variable (WHO-5 score) rather than using the categorical classification, to preserve statistical information and avoid power loss from dichotomization. Odds ratios are presented per 10-unit increase to facilitate interpretation given the 0–100 scale range. From the DAGs, we derived adjustment sets of variables. Different adjustment sets were defined for the logistic regression models of MDA and DAPSA remission. Both models included the following covariates: sex, age, BMI, sports, naive to b/tsDMARD therapy, number of comorbidities, symptom duration joints, and type of inclusion therapy. For the MDA model, we additionally adjusted for the baseline values of the MDA components: TJC, SJC, BSA, PtGA MSK, HAQ, and enthesitis (yes/no). We did not adjust for PtGA, because of its close correlation with the exposure of interest. For the DAPSA remission model, in addition to the common covariates, we adjusted for the baseline DAPSA, as well as variables not part of the DAPSA definition but relevant to disease activity: BSA, HAQ, and enthesitis. To test whether the association between baseline well-being and remission differed by sex, sex was entered as an interaction term with WHO-5 score (rather than as a main effect alone) in each model. Effect modification was assessed from the interaction term’s estimate, 95% CI, and p-value.

To assess the robustness of our findings, we conducted two additional sensitivity analyses. First, we repeated the primary models using objective-only response indicators in place of the composite outcomes: swollen joint count (SJC ≤ 1) in place of MDA, and CRP normalization (CRP < 5 mg/L) in place of DAPSA remission, using the same confounder sets as the corresponding primary models (with SJC additionally tested under DAPSA’s confounder set as a triangulation check). Second, we repeated the primary models restricted to patients with observed baseline WHO-5 scores, rather than the full multiply imputed sample.

We fitted separate regression models for each imputed data set and outcome [33], adjusting for those variables and combining estimates according to Rubin’s rules [34]. Adjusted odds ratios (OR) are given with 95% confidence intervals (95% CI). Calculations were carried out with R, version 4.4.1 (R Foundation for Statistical Computing, Vienna, Austria) [35].

## Results

There were 2,416 PsA patients in our dataset, of whom 277 had not yet reached 6 months of follow-up and were excluded, leaving 2,139 in this analysis. Of these, 251 patients did not have a valid WHO-5 score, primarily because the patient CRF was missing entirely; only 58 of these patients had any patient CRF data available. Baseline characteristics of the 1,888 patients with a valid WHO-5 score are shown in Table 1. Additional file 1: Table S1 additionally includes the 251 patients with unknown WHO-5 status; patient-reported variables for this group are based on small numbers and should be interpreted with caution. All subsequent analyses are based on multiply imputed data from the full analytic sample of 2,139 patients; a comparison of observed and imputed data is provided in Additional file 1: Table S2. Patient flow through the study is shown in Additional file 1: Figure S1 (STROBE diagram).

Of the 1,888 patients with a WHO-5 included in the analysis, 1,108 were women and 780 were men. Moderate or severe depressive symptoms at baseline were more common among women than men (34% vs. 23%).

Patients with moderate/severe depressive symptoms differed from those with no/mild symptoms in a largely consistent pattern across sexes (Table 1). Engagement in sports was less common in both women and men (36% vs. 47% in women and 29% vs. 44% in men), and obesity was somewhat more prevalent (42% vs. 37% in women and 41% vs. 34% in men). Disease activity was markedly higher in patients with moderate/severe depressive symptoms in both sexes. The proportion with ≥ 5 tender joints was higher in both women and men (59% vs. 49% in women and 59% vs. 41% in men), as was the proportion with ≥ 5 swollen joints (30% vs. 21% in women and 34% vs. 23% in men) and the proportion with enthesitis (31% vs. 25% in women and 21% vs. 18% in men). Mean DAPSA scores were approximately 6–7 points higher in both sexes. Comorbidity burden was also higher in patients with moderate/severe depressive symptoms (≥ 3 comorbidities: 45% vs. 33% in women and 34% vs. 27% in men). Glucocorticoid use was more frequent in the moderate/severe group in both sexes (39% vs. 30% in women and 34% vs. 27% in men). All PROs were substantially worse in patients with moderate/severe depressive symptoms, with the largest absolute differences seen for fatigue and PsAID scores.

Two patterns differed notably between sexes. Current smoking was more common among women with moderate/severe symptoms than among those with no/mild symptoms (39% vs. 31%), while in men the rates were virtually identical (26% vs. 25%); women also smoked more than men overall. Similarly, higher education was less frequent among women with moderate/severe symptoms (47% vs. 56%), whereas no such difference was observed in men (55% vs. 53%).

**Table 1:** Baseline characteristics of study participants

	Women		Men			
	None/mild *N* = 736	Moderate/severe *N* = 372	None/mild *N* = 598	Moderate/severe *N* = 182	Total *N* = 1888	Available (%)
**Sociodemographic characteristics**						
Age, mean (SD)	53.6 (12.9)	52.6 (11.3)	51.7 (13.2)	50.8 (11.1)	52.5 (12.6)	100
BMI, mean (SD)	28.7 (6.3)	29.5 (6.7)	28.6 (4.9)	29.8 (5.6)	28.9 (5.9)	98
BMI ≥ 30, n (%)	266 (37)	155 (42)	201 (34)	74 (41)	696 (37)	98
Smoking current, n (%)	215 (31)	140 (39)	142 (25)	45 (26)	542 (30)	96
Education, > 10 years (ISCED 3), n (%)	395 (56)	169 (47)	308 (53)	97 (55)	969 (53)	96
Sports ≥1 h/week, n (%)	330 (47)	132 (36)	258 (44)	53 (29)	773 (42)	97
**Clinical characteristics**						
Symptom duration joints in years, median (Q1-Q3)	7.1 (2.8–14.5)	7.7 (3.1–16.1)	6.1 (2.4–12.1)	5.6 (1.9–12.7)	6.8 (2.6–13.8)	98
Duration since diagnosis joints in years, median (Q1-Q3)	4.5 (1.1–9.4)	4.2 (1.1–10.2)	3.1 (0.7–8.8)	3.4 (0.8–9.9)	3.9 (0.9–9.4)	91
Nail psoriasis, n (%)	249 (34)	139 (38)	282 (48)	83 (46)	753 (40)	100
Body surface area affected by PsO in %, mean (SD)	6.0 (12.2)	7.6 (14.6)	9.2 (14.3)	8.5 (14.9)	7.6 (13.7)	98
Enthesitis current, n (%)	183 (25)	114 (31)	108 (18)	38 (21)	443 (24)	99
Tender joints, n (%)	627 (85)	324 (88)	476 (80)	162 (89)	1,589 (85)	100
≥5 tender joints, n (%)	358 (49)	216 (59)	246 (41)	107 (59)	927 (49)	100
Swollen joints, n (%)	477 (65)	254 (69)	394 (66)	134 (74)	1,259 (67)	100
≥5 swollen joints, n (%)	154 (21)	111 (30)	138 (23)	61 (34)	464 (25)	100
CRP ≥5 mg/L, n (%)	256 (39)	138 (39)	218 (40)	75 (44)	687 (40)	92
≥3 Comorbidities, n (%)	245 (33)	167 (45)	160 (27)	62 (34)	634 (34)	100
PhGA Skin (NRS 0–10), mean (SD)	2.9 (2.5)	3.1 (2.5)	3.5 (2.4)	3.2 (2.4)	3.2 (2.5)	94
PhGA MSK (NRS 0–10), mean (SD)	4.9 (2.0)	5.5 (2.1)	4.6 (2.1)	5.7 (1.8)	5.0 (2.1)	94
DAPSA, mean (SD)	21.1 (12.1)	27.7 (14.3)	19.3 (11.9)	26.2 (12.9)	22.3 (13.0)	91
**Patient-reported characteristics**						
PtGA Skin (NRS 0–10), mean (SD)	3.5 (2.9)	4.5 (3.2)	3.6 (2.8)	4.3 (2.9)	3.8 (2.9)	99
PtGA MSK (NRS 0–10), mean (SD)	5.3 (2.2)	7.0 (1.9)	4.7 (2.4)	6.4 (2.1)	5.6 (2.4)	100
Pain (NRS 0–10), mean (SD)	5.1 (2.2)	7.0 (2.0)	4.6 (2.5)	6.5 (2.1)	5.4 (2.4)	98
Fatigue (NRS 0–10), mean (SD)	4.9 (2.5)	7.5 (1.8)	4.0 (2.5)	6.7 (2.2)	5.3 (2.7)	98
HAQ (0–3), mean (SD)	0.9 (0.6)	1.4 (0.6)	0.6 (0.6)	1.2 (0.6)	0.9 (0.7)	99
PsAID (0–10), mean (SD)	3.8 (1.9)	6.0 (1.9)	3.3 (1.8)	5.6 (2.0)	4.2 (2.2)	93
DLQI (0–30), mean (SD)	4.9 (5.7)	7.5 (7.3)	4.6 (5.1)	7.5 (7.4)	5.6 (6.2)	97
**Treatments**						
B/tsDMARD naÏve, n (%)	443 (60)	206 (55)	402 (67)	94 (52)	1,145 (61)	100
Inclusion therapy, n (%)						100
NSAID	10 (1.4)	4 (1.1)	4 (0.7)	2 (1.1)	20 (1.1)	
csDMARD	122 (17)	50 (13)	106 (18)	20 (11)	298 (16)	
bDMARD	533 (72)	284 (76)	454 (76)	143 (79)	1,414 (75)	
tsDMARD	71 (9.6)	34 (9.1)	34 (5.7)	17 (9.3)	156 (8.3)	
NSAIDs current, n (%)	307 (42)	178 (48)	216 (36)	70 (38)	771 (41)	100
CsDMARDs current, n (%)	333 (45)	145 (39)	279 (47)	66 (36)	823 (44)	100
Glucocorticoids current, n (%)	221 (30)	145 (39)	162 (27)	61 (34)	589 (31)	99

b/tsDMARD, biological/targeted synthetic Disease-Modifying Anti-Rheumatic Drug; BMI, Body Mass Index; CASPAR, ClASsification criteria for Psoriatic ARthritis; CRP, C-reactive protein; csDMARDS, conventional synthetic Disease-Modifying Anti-Rheumatic Drugs; DAPSA, Disease Activity in PSoriatic Arthritis; DLQI, Dermatology Life Quality Index; HAQ, Health Assessment Questionnaire; MSK, musculoskeletal; NRS, Numeric Rating Scale; NSAIDS, Non-Steroidal Anti-Inflammatory Drugs; PhGA, Physician Global Assessment; PsA, Psoriatic Arthritis; PsAID, Psoriatic Arthritis Impact of Disease; PsO, Psoriasis; PtGA, Patient Global Assessment; Q1, first quartile; Q3, third quartile; SD, standard deviation; WHO-5, World Health Organization-5 Well-Being Index. Note: Patients with unknown WHO-5 status were excluded (women: *n* = 153; men: *n* = 98). None/mild and moderate/severe depressive symptoms were defined as WHO-5 score ≥ 29 and < 29 (0–100 scale), respectively

**Fig. 1:**
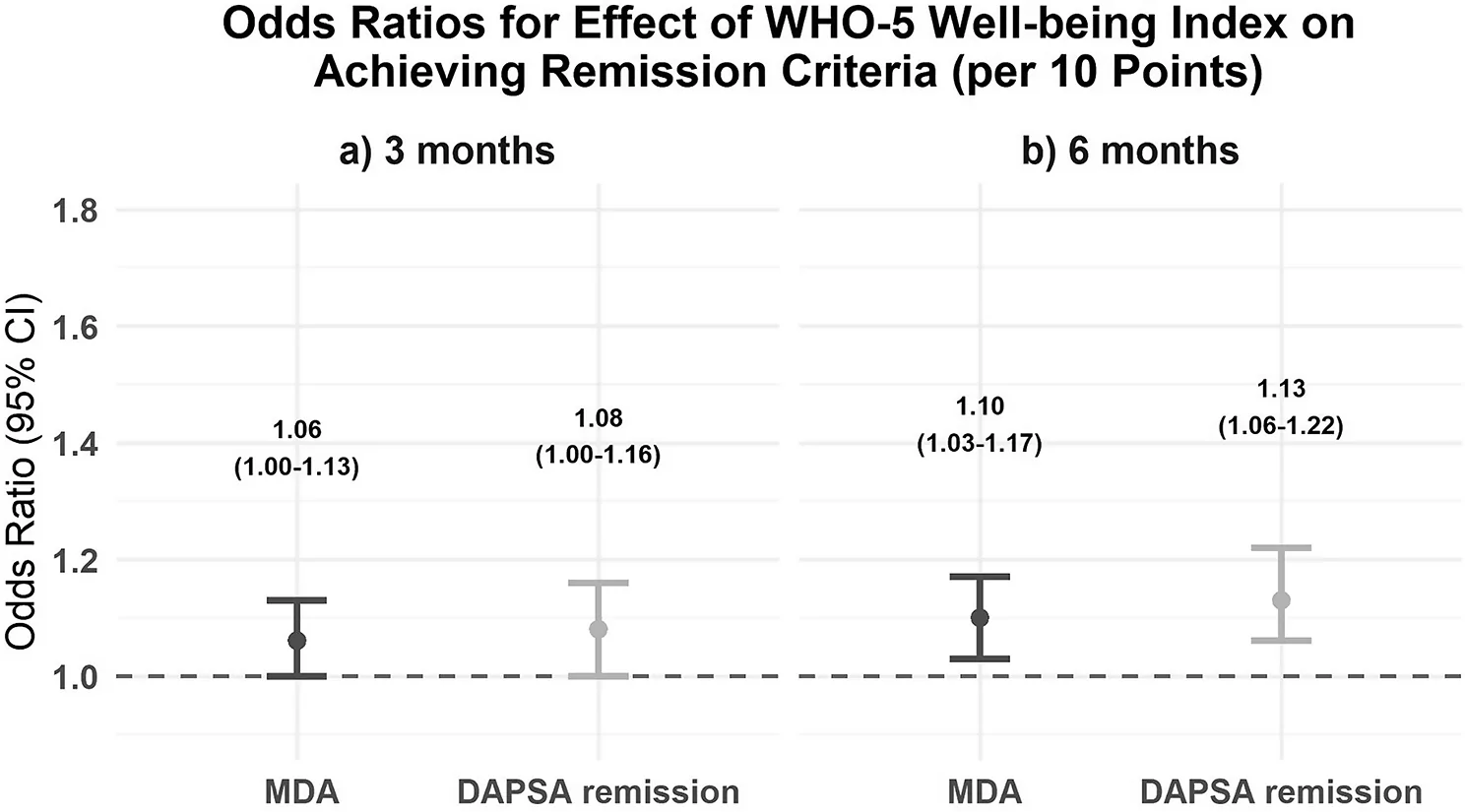
(based on imputed data): Odds ratios for the association between the WHO-5 Well-being Index and reaching MDA or DAPSA remission at 3 and 6 months (per 10 points). (DAPSA, Disease Activity in Psoriatic Arthritis; MDA, Minimal Disease Activity; WHO-5, World Health Organization Five Well-Being Index.)

Figure 1 presents the main results of this analysis (based on imputed data): the logistic regression models examining the association between baseline well-being and achievement of MDA or DAPSA remission at 3 and 6 months. In the total cohort, each 10-point increase in baseline WHO-5 score was significantly associated with higher odds of MDA at 6 months (OR 1.10, 95% CI 1.03–1.17), DAPSA remission at 3 months (OR 1.08, 95% CI 1.00–1.16), and DAPSA remission at 6 months (OR 1.14, 95% CI 1.06–1.22), while the association with MDA at 3 months did not reach statistical significance (OR 1.06, 95% CI 1.00–1.13, *p* = 0.051). To test whether these associations were modified by sex, a sex × WHO-5 interaction term was added to each model. None of the four interaction terms reached statistical significance (MDA at 3 months: OR 1.05, 95% CI 0.94–1.17, *p* = 0.42; MDA at 6 months: OR 1.06, 95% CI 0.95–1.19, *p* = 0.29; DAPSA remission at 3 months: OR 0.98, 95% CI 0.86–1.12, *p* = 0.76; DAPSA remission at 6 months: OR 0.98, 95% CI 0.86–1.12, *p* = 0.77), indicating no evidence that the association between baseline well-being and remission differed between women and men.

In contrast to the composite outcomes, baseline well-being was not associated with SJC-based response at either timepoint (OR 1.00 [0.94–1.07] at 3 months; OR 0.98 [0.92–1.05] at 6 months); this null finding was unchanged when SJC was instead adjusted using DAPSA’s confounder set (OR 1.00 [0.94–1.07] and OR 0.98 [0.92–1.04], respectively), indicating it is not sensitive to the choice of confounder specification. For CRP normalization, the association was also attenuated relative to the composite DAPSA outcome and reversed in direction, reaching significance at 6 months (OR 0.91, 95% CI 0.86–0.97, *p* = 0.0034), indicating lower odds of CRP normalization among patients with higher baseline well-being [Additional file 1: Table S4].

As a further check on the robustness of our findings, we repeated the primary analysis restricted to patients with observed baseline WHO-5 (*n* = 1,888), rather than including those with imputed WHO-5 values. Results were closely consistent with the primary analysis across all four models (MDA at 3 months: OR 1.06 [1.00–1.12] vs. 1.06 [1.00–1.13]; MDA at 6 months: OR 1.10 [1.04–1.17] vs. 1.10 [1.03–1.17]; DAPSA remission at 3 months: OR 1.07 [0.99–1.15], *p* = 0.068, vs. 1.08 [1.00–1.16], *p* = 0.040; DAPSA remission at 6 months: OR 1.14 [1.06–1.22] vs. 1.13 [1.06–1.22]), indicating our results are not driven by imputation of missing WHO-5 values. The slightly attenuated significance for DAPSA remission at 3 months in the complete-case analysis is consistent with reduced statistical power from the smaller sample rather than a systematic difference in effect.

**Fig. 2:**
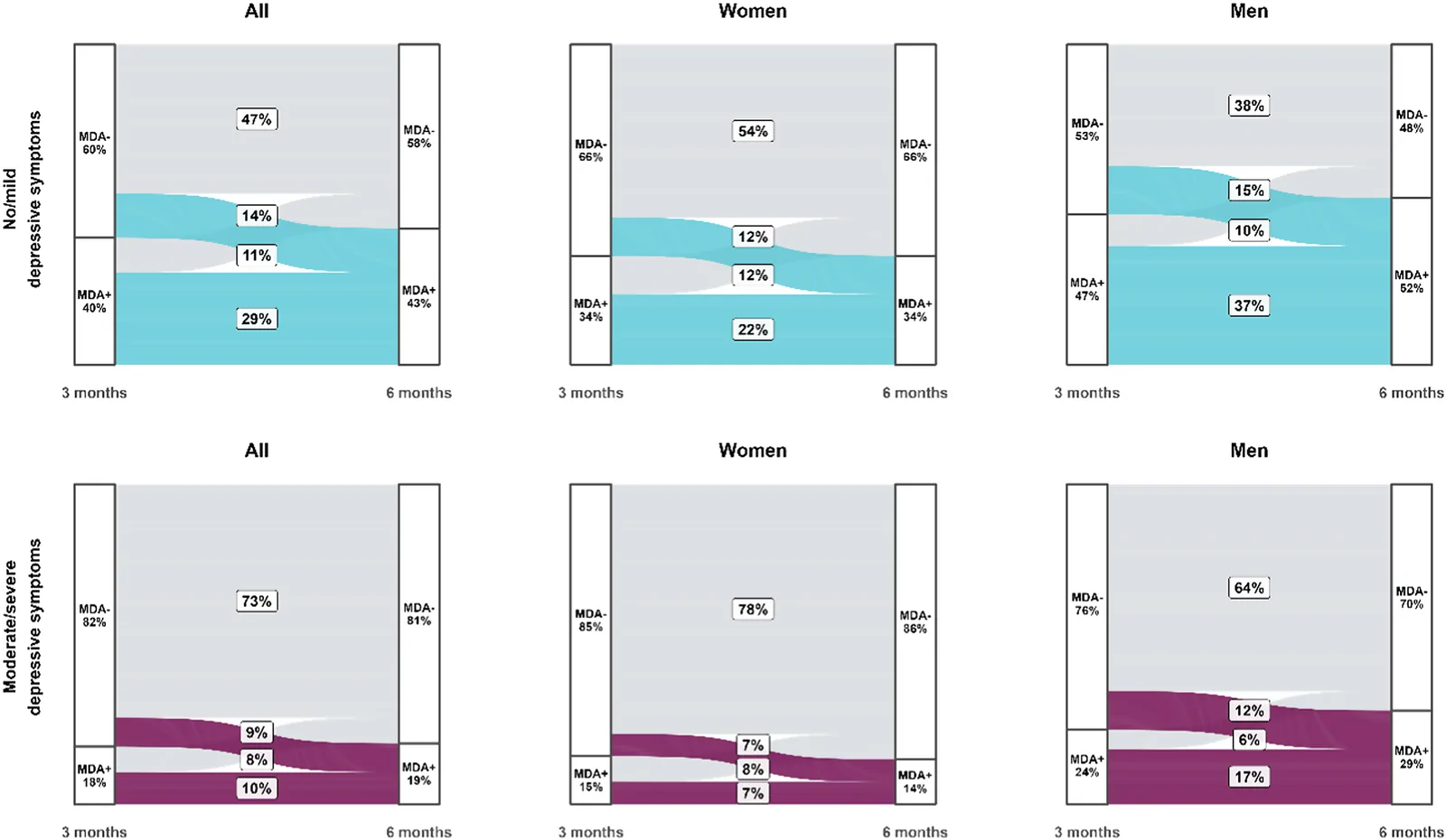
(based on imputed data): Proportion of Patients Achieving MDA by Category of Depressive Symptoms and Time Point (MDA, Minimal Disease Activity. Flow widths represent percentages of the total group number. E.g. 60% of patients with no/mild depressive symptoms do not reach MDA at 3 months; of these, about a quarter (14% of all patients with no/mild depressive symptoms) reach MDA at 6 months. Due to rounding, percentages may not sum to exactly 100%.)

Figure 2 (based on imputed data) illustrates trajectories with respect to MDA over 3 and 6 months. Among patients with no or mild depressive symptoms, 40% achieved MDA at 3 months, and 29% achieved sustained remission (MDA at both time points). An additional 14% who were not in MDA at 3 months achieved MDA by 6 months. In contrast, among patients with moderate/severe depressive symptoms, only 18% achieved MDA at 3 months, 10% achieved sustained remission, and 9% newly achieved MDA between 3 and 6 months.

These patterns were evident in both sexes when examined descriptively. Among women with no/mild depressive symptoms, 34% achieved MDA at 3 months and 22% sustained remission, compared to 15% and 7%, respectively, among women with moderate/severe symptoms; among men with no/mild depressive symptoms, 47% achieved MDA at 3 months and 37% sustained remission, compared to 24% and 17%, respectively, among men with moderate/severe symptoms. Overall MDA rates were lower in women than in men regardless of depressive symptom category; testing for effect modification by sex did not support a differential association between well-being and remission (see below).

The corresponding diagram for DAPSA remission is provided in Additional file 1: Figure S2. It shows broadly similar patterns, with lower proportions of women with depressive symptoms achieving and maintaining DAPSA remission compared to their counterparts with no or mild symptoms, while in men the proportions are somewhat higher but follow the same overall trends.

**Fig. 3:**
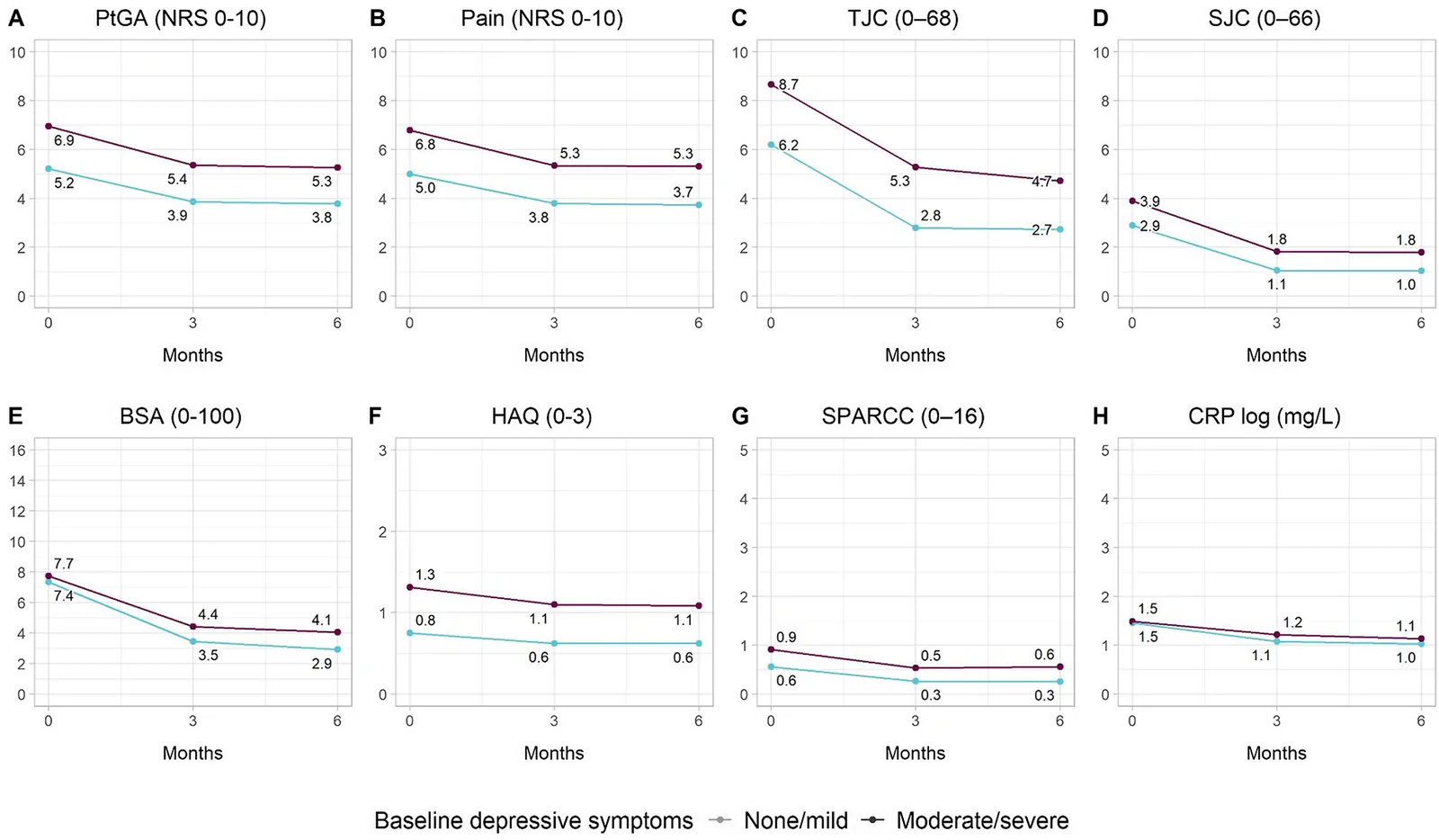
(based on imputed data): Time course of mean component values of DAPSA and MDA at baseline, 3 months, and 6 months, stratified by depressive symptom category. (BSA, Body surface area affected by psoriasis; CRP, C-reactive protein; DAPSA, Disease Activity in Psoriatic Arthritis; HAQ, Health Assessment Questionnaire; MDA, Minimal Disease Activity; NRS, Numeric Rating Scale; PtGA, Patient Global Assessment; SJC, Swollen Joint Count; SPARCC, Spondyloarthritis Research Consortium of Canada enthesitis index; TJC, Tender Joint Count. Panels A–G represent components of MDA. Panels A–D and H represent components of DAPSA.)

Figure 3 (based on imputed data) shows the time course of mean component values of DAPSA and MDA, stratified by depressive symptom category. With the exception of (log-transformed) CRP, patients with moderate/severe depressive symptoms consistently showed higher values compared to those with no/mild symptoms at baseline, 3 months, and 6 months. While patients with moderate/severe depressive symptoms also showed improvement over time, all parameters remained higher than in patients with no/mild symptoms. The corresponding sex-stratified analyses are shown in Additional file 1: Figures S3 and S4. While baseline values differ slightly between women and men, the changes over time are largely comparable.

## Discussion

In this large cohort of patients with PsA, nearly one-third of the patients reported moderate/severe depressive symptoms. These patients had more comorbidities, higher disease activity, and less favorable PROs at baseline. Over follow-up, they were less likely to achieve MDA and DAPSA remission than patients with no/mild depressive symptoms, and differences in individual disease activity components persisted despite improvements in both groups. Logistic regression analyses confirmed an association between baseline well-being and remission, with no evidence that this association was modified by sex.

An important consideration is the extent to which our findings reflect objective improvement in inflammatory disease activity versus patient-reported symptom burden. When we repeated our analyses using objective-only response indicators — swollen joint count (SJC) in place of MDA, and CRP normalization in place of DAPSA remission — baseline well-being was not associated with SJC-based response at either timepoint, and was inversely associated with CRP normalization at 6 months. This indicates that the association we observed between baseline well-being and the composite MDA and DAPSA remission outcomes is predominantly attributable to their patient-reported components (e.g., pain, patient global assessment, function) rather than to objective inflammatory improvement. Our findings should therefore be interpreted as reflecting an association between depressive symptoms and how patients experience and report their overall symptom burden, rather than evidence that depressive symptoms influence the underlying inflammatory disease process.

Our findings are consistent with earlier studies in RA and PsA that have demonstrated an association between depression and worse treatment outcomes. Michelsen et al. [5] found that baseline depression and anxiety, as measured by various criteria, negatively predicted remission after 3 and 6 months of treatment in RA and partly in PsA. Patients with depression and anxiety had lower remission rates across multiple criteria, including Disease Activity Score, Simplified Disease Activity Index, Clinical Disease Activity Index, American College of Rheumatology (ACR) / European Alliance of Associations for Rheumatology Boolean (EULAR boolean), and DAPSA. Martins et al. [10] examined the association between depression and clinical response to bDMARDs in patients with PsA. Patients with depression exhibited significantly lower ACR20/50/70 response rates and worse responses according to EULAR response criteria [36] after 12 months of therapy compared to those without depression. Additionally, patients with depression and axial involvement showed a poorer response based on ASDAS criteria [37]. The rate of switching to another bDMARD due to inefficacy within the first year was also significantly higher in patients with depression. Snoeck Henkemans et al. [6] investigated the association between depression and anxiety and the inability to achieve remission in RA and PsA patients. Depressive symptoms were associated with a lower likelihood of achieving remission over two years of follow-up in both RA and PsA patients, even after adjusting for concurrent anxiety symptoms. Wong et al. [7] found that the presence of depression/anxiety was associated with a reduced probability of achieving sustained MDA in PsA patients, defined as achieving MDA at 2 subsequent visits.

Our results extend previous findings by testing whether the association between well-being and treatment response differs by sex, rather than relying on stratified comparisons alone. Although exploratory subgroup estimates initially appeared larger in women than in men, a sex × WHO-5 interaction term added to each model showed no evidence of effect modification for either outcome at either timepoint. Stratified estimates that differ in statistical significance are not equivalent to a formally demonstrated difference in effect, and can be misleading if interpreted as such without an explicit interaction test. Given the limited and inconsistent reporting of sex-disaggregated results [38, 39], this distinction between stratified comparisons and tested effect modification may be relevant more broadly to studies examining sex differences.

Our findings highlight the clinical importance of systematically assessing depressive symptoms in patients with PsA. Routine use of brief, validated instruments such as the WHO-5 could help identify patients less likely to report symptom-based remission, independent of their underlying inflammatory disease activity. Addressing depressive symptoms through timely referral for psychological or pharmacological interventions may improve mental health; whether this could also improve response to antirheumatic therapy has not been demonstrated by this study and remains to be tested in interventional studies. In Germany, digital health applications such as web-based cognitive behavioral therapy programs (e.g., GET.ON, Deprexis) are approved for treating depressive symptoms and can be prescribed within statutory health insurance [40]. These interventions have demonstrated clinically meaningful reductions in depression severity in implementation studies and meta-analyses [41, 42], potentially offering an accessible adjunct to traditional mental health care for patients with chronic rheumatic diseases.

Future studies should employ repeated measures of both depressive symptoms and disease activity to assess the direction and temporality of their association, for example using time-varying exposure models, rather than the baseline-only exposure used here. Larger, ideally multi-cohort collaborations, such as those coordinated through the EuroSpA consortium, would allow adequately powered testing of effect modification by sex and other patient characteristics, since our null interaction findings do not rule out modest effect modification undetectable at this that this sample size. Longitudinal modeling of disease-activity change overtime, for example using mixed-effects models with a time × depressive symptom group interaction, could complement the component values presented here (Fig. 3) and directly test whether patterns of improvement differ between groups. Mediation analyses could help clarify how much of the well-being–remission association is attributable to inflammatory versus patient-reported symptom domains, addressing the conceptual overlap discussed above. Finally, randomized interventional studies of psychological or pharmacological treatment for depressive symptoms, with PsA disease activity or remission as a secondary endpoint, are needed to establish whether treating depressive symptoms improves treatment response, building on promising evidence from RA [43, 44].

### Strengths and limitations

The main strengths of this analysis are the large sample size and the prospective collection of real-world data, using standardized CRFs across participating centers supported by an automated data monitoring process [12], supplemented by manual review of complex, primarily longitudinal, data issues that cannot be captured through automated checks alone. Sensitivity analyses confirmed that our findings were robust to exclusion of patients with imputed baseline well-being scores. Another strength is the use of a DAG [Additional file 1: Figures S5 and S6] to systematically identify potential confounders, which included baseline disease activity components (specific to each outcome — see Methods), BMI, comorbidities, therapy type, and symptom duration.

Several limitations must be considered. Depression was not formally diagnosed; we used the WHO-5, a validated screening instrument, rather than documented comorbid depression, as earlier analyses indicated substantial underreporting of this diagnosis even among patients with severe depressive symptoms [4]. Our MDA definition was also adapted from the standard criteria to match available registry data: body surface area (BSA) was used in place of PASI, and NRS-based cut-offs were used in place of VAS-based cut-offs for the patient-reported components. This adaptation has not been separately validated, which should be considered when interpreting MDA-based results. Our research question focused on baseline depressive symptoms and treatment response, not changes in depressive symptoms over time, although the relationship between depressive symptoms and disease activity is likely bidirectional. The validity of our results depends on the correctness of the underlying DAG and statistical model; misspecification, such as omitted variables or incorrect assumptions about causal pathways, could bias the estimates. Furthermore, patients with moderate/severe depressive symptoms differed from those with no/mild symptoms across multiple baseline characteristics, including higher disease activity, comorbidity burden, BMI, and glucocorticoid use, and lower physical activity (Table 1). Despite adjustment for DAG-identified confounders, we cannot exclude confounding by indication; our results should be interpreted as reflecting this less favorable baseline profile rather than a specific causal treatment-response mechanism. There is also considerable conceptual overlap between the WHO-5 and several patient-reported components of MDA and DAPSA — including pain, PtGA, fatigue, and HAQ. A sensitivity analysis using objective-only outcome components (SJC-based response in place of MDA; CRP normalization in place of DAPSA remission; see Results) found no association between well-being and SJC-based response, and an inverse association with CRP normalization at 6 months, indicating the composite-outcome association is predominantly attributable to patient-reported components rather than objective inflammatory improvement. Patients with unknown WHO-5 status (excluded from analyses using observed data, but retained after multiple imputation) were broadly similar to patients with known WHO-5 status on baseline characteristics with near-complete availability (age, BMI, joint counts, CRP, comorbidity burden, and treatment; see Additional file 1: Table S1); larger apparent differences were confined to sociodemographic and patient-reported variables that were themselves largely missing for this group, due to shared reliance on the patient questionnaire, and we therefore do not interpret these as evidence of a systematically different population. Despite adjustment for identified confounders, residual confounding due to unmeasured or imperfectly measured variables cannot be excluded, which may limit the robustness and generalizability of our findings.

In summary, depressive symptoms were common in PsA and associated with higher disease activity, less frequent achievement of MDA and DAPSA remission, and poorer outcomes over time, regardless of sex. This suggests that mental health should be considered as an integral component of PsA management and that routine screening for depressive symptoms may be warranted in clinical practice.

## Supplementary Information

Below is the link to the electronic supplementary material.

Supplementary Material 1

## Data Availability

The data that support the findings of this study are available from German Rheumatology Research Center but restrictions apply to the availability of these data, which were used under license for the current study, and so are not publicly available. Data are however available from the authors upon reasonable request and with permission of the German Rheumatology Research Center.

## Abbreviations

ACR: American college of rheumatology
ASAS: Assessment of spondyloarthritis international society
ASDAS: Ankylosing spondylitis disease activity score
b/tsDMARD: biological/targeted synthetic disease-modifying anti-rheumatic drug
BMI: Body mass index
BSA: Body surface area
CASPAR: ClASsification criteria for psoriatic arthritis
CI: Confidence interval
CRF: Case report form
CRP: C-reactive protein
DAG: Directed acyclic graph
DAPSA: Disease activity in psoriatic arthritis
DLQI: Dermatology life quality index
EULAR: European alliance of associations for rheumatology
FMI: Fraction of missing information
HAQ(-DI): Health assessment questionnaire (Disability Index)
ISCED: International standard classification of education
m: number of imputations
MAR: Missing at random
maxit: maximum iterations
MDA: Minimal disease activity
MICE: Multivariate imputation by chained equations
MSK: Musculoskeletal
NRS: Numeric rating scale
NSAID(s): Non-steroidal anti-inflammatory drug(s)
OMERACT: Outcome measures in rheumatology
OR: Odds ratio
PASI: Psoriasis area and severity index
PhGA: Physician global assessment
PMM: Predictive mean matching
PsA: Psoriatic arthritis
PsAID: Psoriatic Arthritis impact of disease
PsO: Psoriasis
PtGA: Patient global assessment
Q1, Q3: First, third quartile
RA: Rheumatoid arthritis
SD: Standard deviation
SJC: Swollen joint count
SPARCC: Spondyloarthritis research consortium of canada
STROBE: STrengthening the reporting of observational studies in epidemiology
TJC: Tender joint count
VAS: Visual analogue scale
WHO-5: World health organization-5 well-being index

## References

[1] FitzGerald O, Ogdie A, Chandran V, Coates LC, Kavanaugh A, Tillett W, et al. Psoriatic arthritis. Nat Reviews Disease Primers. 2021;7(1):59. 10.1038/s41572-021-00293-y 34385474

[2] Gupta S, Syrimi Z, Hughes DM, Zhao SS. Comorbidities in psoriatic arthritis: a systematic review and meta-analysis. Rheumatol Int. 2021;41(2):275–84. 10.1007/s00296-020-04775-2 PMC7835184 33423070

[3] Zhao SS, Miller N, Harrison N, Duffield SJ, Dey M, Goodson NJ. Systematic review of mental health comorbidities in psoriatic arthritis. Clin Rheumatol. 2020;39(1):217–25. 10.1007/s10067-019-04734-8 31486931

[4] Reich A, Weiß A, Lindner L, Baraliakos X, Poddubnyy D, Zinke S, et al. Depressive symptoms are associated with fatigue, poorer functional status and less engagement in sports in axSpA and PsA: an analysis from the RABBIT-SpA cohort. Arthritis Res Ther. 2023;25(1):136. 10.1186/s13075-023-03127-2 PMC10394807 37533077

[5] Michelsen B, Kristianslund EK, Sexton J, Hammer HB, Fagerli KM, Lie E, et al. Do depression and anxiety reduce the likelihood of remission in rheumatoid arthritis and psoriatic arthritis? Data from the prospective multicentre NOR-DMARD study. Ann Rheum Dis. 2017;76(11):1906–10. 10.1136/annrheumdis-2017-211284 28733473

[6] Snoeck Henkemans SVJ, Vis M, Koc GH, Luime JJ, Kok MR, Tchetverikov I, et al. Association between depression and anxiety and inability to achieve remission in rheumatoid arthritis and psoriatic arthritis. Rheumatology (Oxford). 2025;64(5):2411–21. 10.1093/rheumatology/keae621 PMC12048054 39504461

[7] Wong A, Ye JY, Cook RJ, Gladman DD, Chandran V. Depression and Anxiety Reduce the Probability of Achieving a State of Sustained Minimal Disease Activity in Patients With Psoriatic Arthritis. Arthritis Care Res (Hoboken). 2022;74(9):1430–4. 10.1002/acr.24593 33662181

[8] Reich A, Weiß A, Lindner L, Zinke S, Stille C, Detert J, et al. Effect of depressive symptoms on treatment response in patients with axSpA: data from the RABBIT-SpA register. RMD Open. 2025;11(2):e005422. 10.1136/rmdopen-2025-005422 PMC12067848 40345709

[9] Zhao SS, Jones GT, Hughes DM, Moots RJ, Goodson NJ. Depression and anxiety symptoms at TNF inhibitor initiation are associated with impaired treatment response in axial spondyloarthritis. Rheumatology (Oxford). 2021;60(12):5734–42. 10.1093/rheumatology/keab242 PMC8645272 33713118

[10] Martins A, Oliveira D, Nicolau R, Rocha TM, Bernardo A, Costa L, et al. What is the association of depression with clinical response to therapy in patients with psoriatic arthritis treated with biologic disease-modifying antirheumatic drugs? Clin Rheumatol. 2024;43(1):251–8. 10.1007/s10067-023-06806-2 37957488

[11] Lindner L, Weiß A, Reich A, Baumann C, Behrens F, Baraliakos X, et al. Real-world sex differences in treatment persistence and reasons for discontinuation in psoriatic arthritis patients: results from the German RABBIT-SpA register. Arthritis Res Ther. 2025;27(1):188. 10.1186/s13075-025-03650-4 PMC12490096 41039496

[12] Lindner L, Weiß A, Reich A, Kindler S, Behrens F, Braun J, et al. Implementing an automated monitoring process in a digital, longitudinal observational cohort study. Arthritis Res Ther. 2021;23(1):181. 10.1186/s13075-021-02563-2 PMC8262053 34233730

[13] Regierer AC, Weiß A, Baraliakos X, Zink A, Listing J, Strangfeld A. [RABBIT-SpA: a new disease register for axial spondyloarthritis and psoriatic arthritis]. Z Rheumatol. 2020;79(2):135–42. 10.1007/s00393-019-0613-z 30874933

[14] UNESCO Institute for Statistics. International standard classification of education: ISCED 2011. Montreal: UNESCO Institute for Statistics; 2012.

[15] Taylor W, Gladman D, Helliwell P, Marchesoni A, Mease P, Mielants H. Classification criteria for psoriatic arthritis: development of new criteria from a large international study. Arthritis Rheum. 2006;54(8):2665–73. 10.1002/art.21972 16871531

[16] Maksymowych WP, Mallon C, Morrow S, Shojania K, Olszynski WP, Wong RL, et al. Development and validation of the Spondyloarthritis Research Consortium of Canada (SPARCC) Enthesitis Index. Ann Rheum Dis. 2009;68(6):948–53. 10.1136/ard.2007.084244 18524792

[17] Cauli A, Gladman DD, Mathieu A, Olivieri I, Porru G, Tak PP, et al. Physician’s Global Assessment in Psoriatic Arthritis: A Multicenter GRAPPA Study. J Rheumatol. 2018;45(9):1256–62. 10.3899/jrheum.171183 29907666

[18] Cauli A, Gladman DD, Mathieu A, Olivieri I, Porru G, Tak PP, et al. Patient global assessment in psoriatic arthritis: a multicenter GRAPPA and OMERACT study. J Rheumatol. 2011;38(5):898–903. 10.3899/jrheum.100857 21324965

[19] Gossec L, de Wit M, Kiltz U, Braun J, Kalyoncu U, Scrivo R, et al. A patient-derived and patient-reported outcome measure for assessing psoriatic arthritis: elaboration and preliminary validation of the Psoriatic Arthritis Impact of Disease (PsAID) questionnaire, a 13-country EULAR initiative. Ann Rheum Dis. 2014;73(6):1012–9. 10.1136/annrheumdis-2014-205207 24790067

[20] Husted JA, Gladman DD, Long JA, Farewell VT. A modified version of the Health Assessment Questionnaire (HAQ) for psoriatic arthritis. Clin Exp Rheumatol. 1995;13(4):439–43. 7586774

[21] Finlay AY, Khan GK. Dermatology Life Quality Index (DLQI) - a simple practical measure for routine clinical use. Clin Exp Dermatol. 1994;19(3):210–6. 10.1111/j.1365-2230.1994.tb01167.x 8033378

[22] Schoels M, Aletaha D, Funovits J, Kavanaugh A, Baker D, Smolen JS. Application of the DAREA/DAPSA score for assessment of disease activity in psoriatic arthritis. Ann Rheum Dis. 2010;69(8):1441–7. 10.1136/ard.2009.122259 20525844

[23] Coates LC, Fransen J, Helliwell PS. Defining minimal disease activity in psoriatic arthritis: a proposed objective target for treatment. Ann Rheum Dis. 2010;69(1):48–53. 10.1136/ard.2008.102053 19147615

[24] Topp CW, Østergaard SD, Søndergaard S, Bech P. The WHO-5 Well-Being Index: A Systematic Review of the Literature. Psychother Psychosom. 2015;84(3):167–76. 10.1159/000376585 25831962

[25] Halliday JA, Hendrieckx C, Busija L, Browne JL, Nefs G, Pouwer F, et al. Validation of the WHO-5 as a first-step screening instrument for depression in adults with diabetes: Results from Diabetes MILES – Australia. Diabetes Res Clin Pract. 2017;132:27–35. 10.1016/j.diabres.2017.07.005 28783530

[26] Löwe B, Spitzer RL, Gräfe K, Kroenke K, Quenter A, Zipfel S, et al. Comparative validity of three screening questionnaires for DSM-IV depressive disorders and physicians’ diagnoses. J Affect Disord. 2004;78(2):131–40. 10.1016/s0165-0327(02)00237-9 14706723

[27] Redeker I, Hoffmann F, Callhoff J, Haibel H, Sieper J, Zink A, et al. Determinants of psychological well-being in axial spondyloarthritis: an analysis based on linked claims and patient-reported survey data. Ann Rheum Dis. 2018;77(7):1017–24. 10.1136/annrheumdis-2017-212629 PMC6029638 29525776

[28] Schoels MM, Aletaha D, Alasti F, Smolen JS. Disease activity in psoriatic arthritis (PsA): defining remission and treatment success using the DAPSA score. Ann Rheum Dis. 2016;75(5):811–8. 10.1136/annrheumdis-2015-207507 26269398

[29] van Buuren S. Flexible Imputation of Missing Data. 2nd ed. Boca Raton, FL: Chapman & Hall/CRC; 2018.

[30] White IR, Royston P, Wood AM. Multiple imputation using chained equations: Issues and guidance for practice. Stat Med. 2011;30(4):377–99. 10.1002/sim.4067 21225900

[31] van Buuren S, Groothuis-Oudshoorn K. mice: Multivariate Imputation by Chained Equations in R. J Stat Softw. 2011;45(3):1–67.

[32] Orbai AM, de Wit M, Mease PJ, Callis Duffin K, Elmamoun M, Tillett W, et al. Updating the Psoriatic Arthritis (PsA) Core Domain Set: A Report from the PsA Workshop at OMERACT 2016. J Rheumatol. 2017;44(10):1522–8. 10.3899/jrheum.160904 PMC5538953 28148697

[33] van Buuren S. Multiple imputation of discrete and continuous data by fully conditional specification. Stat Methods Med Res. 2007;16(3):219–42. 10.1177/0962280206074463 17621469

[34] Rubin DB. Multiple imputation for nonresponse in surveys. New York: John Wiley & Sons, Inc.; 1987.

[35] Team RC. R: A Language and Environment for Statistical Computing. Vienna, Austria: R Foundation for Statistical Computing; 2024.

[36] Fransen J, van Riel PLCM. The Disease Activity Score and the EULAR Response Criteria. Rheumatic Disease Clin. 2009;35(4):745–57. 10.1016/j.rdc.2009.10.001 19962619

[37] Machado P, Landewé R, Lie E, Kvien TK, Braun J, Baker D, et al. Ankylosing Spondylitis Disease Activity Score (ASDAS): defining cut-off values for disease activity states and improvement scores. Ann Rheum Dis. 2011;70(1):47–53. 10.1136/ard.2010.138594 21068095

[38] Coates LC, van der Horst-Bruinsma IE, Lubrano E, Beaver S, Drane E, Ufuktepe B, et al. Sex-Specific Differences in Patients With Psoriatic Arthritis: A Systematic Review. J Rheumatol. 2023;50(4):488–96. 10.3899/jrheum.220386 36243418

[39] Eder L, Mylvaganam S, Pardo Pardo J, Petkovic J, Strand V, Mease P, et al. Sex-related differences in patient characteristics, and efficacy and safety of advanced therapies in randomised clinical trials in psoriatic arthritis: a systematic literature review and meta-analysis. Lancet Rheumatol. 2023;5(12):e716–27. 10.1016/S2665-9913(23)00264-3 38251562

[40] Sippli K, Deckert S, Schmitt J, Scheibe M. Healthcare effects and evidence robustness of reimbursable digital health applications in Germany: a systematic review. NPJ Digit Med. 2025;8(1):495. 10.1038/s41746-025-01879-6 PMC12317029 40750660

[41] Twomey C, O’Reilly G, Bültmann O, Meyer B. Effectiveness of a tailored, integrative Internet intervention (deprexis) for depression: Updated meta-analysis. PLoS ONE. 2020;15(1):e0228100. 10.1371/journal.pone.0228100 PMC6992171 31999743

[42] Etzelmueller A, Heber E, Horvath H, Radkovsky A, Lehr D, Ebert DD. The Evaluation of the GET.ON Nationwide Web-Only Treatment Service for Depression- and Stress-Related Symptoms: Naturalistic Trial. J Med Internet Res. 2024;26:e42976. 10.2196/42976 PMC10870208 38300701

[43] Sharpe L, Sensky T, Timberlake N, Ryan B, Brewin CR, Allard S. A blind, randomized, controlled trial of cognitive-behavioural intervention for patients with recent onset rheumatoid arthritis: preventing psychological and physical morbidity. Pain. 2001;89(2–3):275–83. 10.1016/s0304-3959(00)00379-1 11166484

[44] Zautra AJ, Davis MC, Reich JW, Nicassario P, Tennen H, Finan P, et al. Comparison of cognitive behavioral and mindfulness meditation interventions on adaptation to rheumatoid arthritis for patients with and without history of recurrent depression. J Consult Clin Psychol. 2008;76(3):408–21. 10.1037/0022-006X.76.3.408 18540734

