# Supplementary material for: Depressive symptoms are associated with achieving remission in PsA patients – an analysis from the RABBIT-SpA register
Source: BMC Rheumatol. 2026 Oct 14;10:79. doi: 10.1186/s41927-026-00707-y (PMC13670693; doi:10.1186/s41927-026-00707-y)
Supplement: Supplementary file 1 [file 41927_2026_707_MOESM1_ESM.docx]

**Supplementary Material**

**Table of Contents**

**Table S1: Baseline Characteristics by Depressive Symptoms (Including Unknown WHO-5 Status)**

**Table S2: Comparison of Original and Imputed Data by Depressive Symptoms Category**

**Table S3: Multiple Imputation Diagnostic Summary**

**Table S4: Sensitivity analysis: composite (MDA/DAPSA) vs. objective (SJC/CRP) outcomes**

**Figure S1: Flow of psoriatic arthritis (PsA) patients from the RABBIT-SpA register into the analysis of depressive symptoms and treatment response.**

**Figure S2: Proportion of Patients Achieving DAPSA Remission by Category of Depressive Symptoms, Time Point and Sex**

**Figure S3: Mean values of the individual components of DAPSA and MDA at baseline, 3 months, and 6 months, stratified by depressive symptom category (Women)**

**Figure S4: Mean values of the individual components of DAPSA and MDA at baseline, 3 months, and 6 months, stratified by depressive symptom category (Men)**

**Figure S5: DAG: Direct Effect of WHO-5 Well-being Index on Achieving MDA**

**Figure S6: DAG: Direct Effect of WHO-5 Well-being Index on Achieving DAPSA Remission**

**Abbreviations**

**Table S1** Baseline Characteristics by Depressive Symptoms, Including Patients with Unknown WHO-5 Status (based on observed data)

|  | **Women** | | | **Men** | | |  | |
| --- | --- | --- | --- | --- | --- | --- | --- | --- |
|  | **None/mild** N = 736 | **Moderate/severe** N = 372 | **Unknown** N = 153 | **None/mild** N = 598 | **Moderate/severe** N = 182 | **Unknown** N = 98 | **Total** N = 2139 | **Available (%)** |
| **Sociodemographic characteristics** |  |  |  |  |  |  |  |  |
| Age, mean (SD) | 53.6 (12.9) | 52.6 (11.3) | 52.2 (12.7) | 51.7 (13.2) | 50.8 (11.1) | 50.3 (12.1) | 52.4 (12.6) | 100 |
| BMI, mean (SD) | 28.7 (6.3) | 29.5 (6.7) | 29.9 (6.7) | 28.6 (4.9) | 29.8 (5.6) | 28.8 (5.3) | 29.0 (6.0) | 98 |
| BMI ≥30, n (%) | 266 (37) | 155 (42) | 63 (43) | 201 (34) | 74 (41) | 33 (34) | 792 (38) | 98 |
| Smoking current, n (%) | 215 (31) | 140 (39) | 7 (23) | 142 (25) | 45 (26) | 4 (25) | 553 (30) | 87 |
| Education, > 10 years (ISCED 3), n (%) | 395 (56) | 169 (47) | 11 (38) | 308 (53) | 97 (55) | 7 (50) | 987 (53) | 87 |
| Sports ≥1h/week, n (%) | 330 (47) | 132 (36) | 10 (34) | 258 (44) | 53 (29) | 8 (50) | 791 (42) | 88 |
| **Clinical characteristics** |  |  |  |  |  |  |  |  |
| Symptom duration joints in years, median (Q1-Q3) | 7.1 (2.8-14.5) | 7.7 (3.1-16.1) | 5.3 (2.0-11.2) | 6.1 (2.4-12.1) | 5.6 (1.9-12.7) | 4.9 (1.9-12.2) | 6.6 (2.5-13.5) | 98 |
| Duration since diagnosis joints in years, median (Q1-Q3) | 4.5 (1.1-9.4) | 4.2 (1.1-10.2) | 3.2 (0.8-6.9) | 3.1 (0.7-8.8) | 3.4 (0.8-9.9) | 3.0 (0.3-7.8) | 3.8 (0.9-9.1) | 91 |
| Nail psoriasis, n (%) | 249 (34) | 139 (38) | 40 (27) | 282 (48) | 83 (46) | 43 (44) | 836 (39) | 99 |
| Body surface area affected by PsO in %, mean (SD) | 6.0 (12.2) | 7.6 (14.6) | 6.0 (11.9) | 9.2 (14.3) | 8.5 (14.9) | 8.9 (13.0) | 7.5 (13.6) | 98 |
| Enthesitis current, n (%) | 183 (25) | 114 (31) | 43 (29) | 108 (18) | 38 (21) | 24 (25) | 510 (24) | 99 |
| Tender joints, n (%) | 627 (85) | 324 (88) | 132 (87) | 476 (80) | 162 (89) | 76 (78) | 1,797 (84) | 100 |
| ≥5 tender joints, n (%) | 358 (49) | 216 (59) | 73 (48) | 246 (41) | 107 (59) | 35 (36) | 1,035 (49) | 100 |
| Swollen joints, n (%) | 477 (65) | 254 (69) | 95 (63) | 394 (66) | 134 (74) | 50 (51) | 1,404 (66) | 100 |
| ≥5 swollen joints, n (%) | 154 (21) | 111 (30) | 36 (24) | 138 (23) | 61 (34) | 19 (19) | 519 (24) | 100 |
| CRP ≥5mg/L, n (%) | 256 (39) | 138 (39) | 54 (38) | 218 (40) | 75 (44) | 37 (38) | 778 (39) | 92 |
| ≥3 Comorbidities, n (%) | 245 (33) | 167 (45) | 44 (29) | 160 (27) | 62 (34) | 26 (27) | 704 (33) | 100 |
| PhGA Skin (NRS 0-10), mean (SD) | 2.9 (2.5) | 3.1 (2.5) | 2.7 (2.5) | 3.5 (2.4) | 3.2 (2.4) | 3.1 (2.2) | 3.1 (2.5) | 94 |
| PhGA MSK (NRS 0-10), mean (SD) | 4.9 (2.0) | 5.5 (2.1) | 4.6 (2.2) | 4.6 (2.1) | 5.7 (1.8) | 4.0 (2.3) | 4.9 (2.1) | 94 |
| DAPSA, mean (SD) | 21.1 (12.1) | 27.7 (14.3) | 29.1 (18.1) | 19.3 (11.9) | 26.2 (12.9) | 19.4 (12.1) | 22.4 (13.1) | 83 |
| **Patient-reported characteristics** |  |  |  |  |  |  |  |  |
| PtGA Skin (NRS 0-10), mean (SD) | 3.5 (2.9) | 4.5 (3.2) | 3.3 (3.0) | 3.6 (2.8) | 4.3 (2.9) | 2.7 (3.1) | 3.8 (2.9) | 90 |
| PtGA MSK (NRS 0-10), mean (SD) | 5.3 (2.2) | 7.0 (1.9) | 6.1 (2.1) | 4.7 (2.4) | 6.4 (2.1) | 3.7 (2.7) | 5.6 (2.4) | 91 |
| Pain (NRS 0-10), mean (SD) | 5.1 (2.2) | 7.0 (2.0) | 5.7 (2.3) | 4.6 (2.5) | 6.5 (2.1) | 3.3 (2.5) | 5.4 (2.4) | 89 |
| Fatigue (NRS 0-10), mean (SD) | 4.9 (2.5) | 7.5 (1.8) | 5.9 (2.5) | 4.0 (2.5) | 6.7 (2.2) | 4.1 (3.1) | 5.3 (2.7) | 89 |
| HAQ (0-3), mean (SD) | 0.9 (0.6) | 1.4 (0.6) | 1.0 (0.6) | 0.6 (0.6) | 1.2 (0.6) | 0.6 (0.7) | 0.9 (0.7) | 89 |
| PsAID (0-10), mean (SD) | 3.8 (1.9) | 6.0 (1.9) | 4.6 (2.2) | 3.3 (1.8) | 5.6 (2.0) | 2.9 (2.4) | 4.2 (2.2) | 84 |
| DLQI (0-30), mean (SD) | 4.9 (5.7) | 7.5 (7.3) | 4.7 (5.6) | 4.6 (5.1) | 7.5 (7.4) | 3.5 (3.8) | 5.5 (6.2) | 88 |
| **Treatments** |  |  |  |  |  |  |  |  |
| B/tsDMARD naÏve, n (%) | 443 (60) | 206 (55) | 84 (55) | 402 (67) | 94 (52) | 68 (69) | 1,297 (61) | 100 |
| Inclusion therapy, n (%) |  |  |  |  |  |  |  | 100 |
| NSAID | 10 (1.4) | 4 (1.1) | 3 (2.0) | 4 (0.7) | 2 (1.1) | 1 (1.0) | 24 (1.1) |  |
| csDMARD | 122 (17) | 50 (13) | 17 (11) | 106 (18) | 20 (11) | 13 (13) | 328 (15) |  |
| bDMARD | 533 (72) | 284 (76) | 118 (77) | 454 (76) | 143 (79) | 75 (77) | 1,607 (75) |  |
| tsDMARD | 71 (9.6) | 34 (9.1) | 15 (9.8) | 34 (5.7) | 17 (9.3) | 9 (9.2) | 180 (8.4) |  |
| NSAIDs current, n (%) | 307 (42) | 178 (48) | 69 (45) | 216 (36) | 70 (38) | 36 (37) | 876 (41) | 100 |
| CsDMARDs current, n (%) | 333 (45) | 145 (39) | 65 (42) | 279 (47) | 66 (36) | 33 (34) | 921 (43) | 100 |
| Glucocorticoids current, n (%) | 221 (30) | 145 (39) | 44 (29) | 162 (27) | 61 (34) | 24 (24) | 657 (31) | 99 |
| b/tsDMARD, biological/targeted synthetic Disease-Modifying Anti-Rheumatic Drug; BMI, Body Mass Index; CASPAR, ClASsification criteria for Psoriatic ARthritis; CRP, C-reactive protein; csDMARDS, conventional synthetic Disease-Modifying Anti-Rheumatic Drugs; DAPSA, Disease Activity in PSoriatic Arthritis; DLQI, Dermatology Life Quality Index; HAQ, Health Assessment Questionnaire; MSK, musculoskeletal; NRS, Numeric Rating Scale; NSAIDS, Non-Steroidal Anti-Inflammatory Drugs; PhGA, Physician Global Assessment; PsA, Psoriatic Arthritis; PsAID, Psoriatic Arthritis Impact of Disease; PsO, Psoriasis; PtGA, Patient Global Assessment; Q1, first quartile; Q3, third quartile; SD, standard deviation; WHO-5, World Health Organization-5 Well-Being Index. Note: The 'Unknown' category includes 153 women (40 with questionnaire data) and 98 men (18 with questionnaire data). Percentages in Unknown columns are based on available data only. None/mild and moderate/severe depressive symptoms were defined as WHO-5 score ≥29 and <29 (0–100 scale), respectively. | | | | | | | | |

**Table S2** Comparison of Original and Imputed Data by Depressive Symptoms Category

|  |  | **None/mild** | | **Moderate/severe** | | **Total** | |
| --- | --- | --- | --- | --- | --- | --- | --- |
|  | **Time** | **Original** | **Imputed** | **Original** | **Imputed** | **Original** | **Imputed** |
| **Sociodemographic characteristics** |  |  |  |  |  |  |  |
| Women, n (%) |  | 736 (55) | 840.9 (55) | 372 (67) | 420.1 (68) | 1108 (59) | 1261.0 (59) |
| Age (years), mean (SD) |  | 52.8 (13.1) | 52.6 (13.0) | 52.0 (11.2) | 52.0 (11.4) | 52.5 (12.6) | 52.4 (12.6) |
| BMI (kg/m²), mean (SD) |  | 28.6 (5.7) | 28.7 (5.7) | 29.6 (6.4) | 29.7 (6.4) | 28.9 (5.9) | 29.0 (6.0) |
| BMI ≥30, n (%) |  | 467 (36) | 539.2 (36) | 229 (42) | 263.7 (43) | 696 (37) | 802.9 (38) |
| Smoking current, n (%) |  | 357 (28) | 424.4 (28) | 185 (34) | 210.0 (34) | 542 (30) | 634.4 (30) |
| Education, > 10 years (ISCED 3), n (%) |  | 703 (55) | 829.6 (55) | 266 (49) | 304.1 (49) | 969 (53) | 1133.7 (53) |
| Sports ≥1h/week, n (%) |  | 588 (46) | 689.7 (45) | 185 (34) | 212.5 (34) | 773 (42) | 902.2 (42) |
| **Clinical characteristics** |  |  |  |  |  |  |  |
| Symptom duration joints in years |  |  |  |  |  |  |  |
| mean (SD) |  | 9.7 (9.8) | 9.5 (9.7) | 10.1 (9.7) | 10.0 (9.6) | 9.8 (9.7) | 9.6 (9.7) |
| median (Q1-Q3) |  | 6.7 (2.6-13.3) | 6.5 (2.5-13.2) | 7.2 (2.8-14.4) | 7.1 (2.8-14.2) | 6.8 (2.6-13.8) | 6.6 (2.6-13.5) |
| Body surface area affected by PsO in %, mean (SD) | T0 | 7.4 (13.3) | 7.4 (13.1) | 7.9 (14.7) | 7.7 (14.5) | 7.6 (13.7) | 7.5 (13.5) |
| Body surface area affected by PsO in %, mean (SD) | T1 | 3.4 (7.5) | 3.5 (7.6) | 4.4 (9.8) | 4.4 (9.7) | 3.7 (8.3) | 3.7 (8.3) |
| Body surface area affected by PsO in %, mean (SD) | T2 | 3.0 (7.6) | 2.9 (7.5) | 4.1 (9.5) | 4.1 (9.5) | 3.3 (8.2) | 3.2 (8.1) |
| Enthesitis SPARCC (0-16) | T0 |  |  |  |  |  |  |
| mean (SD) |  | 0.6 (1.5) | 0.6 (1.5) | 0.9 (2.0) | 0.9 (2.0) | 0.7 (1.7) | 0.7 (1.7) |
| median (Q1-Q3) |  | 0.0 (0.0-0.0) | 0.0 (0.0-0.0) | 0.0 (0.0-1.0) | 0.0 (0.0-1.0) | 0.0 (0.0-0.0) | 0.0 (0.0-0.0) |
| Enthesitis SPARCC (0-16) | T1 |  |  |  |  |  |  |
| mean (SD) |  | 0.2 (1.1) | 0.3 (1.1) | 0.6 (1.7) | 0.5 (1.6) | 0.3 (1.3) | 0.3 (1.3) |
| median (Q1-Q3) |  | 0.0 (0.0-0.0) | 0.0 (0.0-0.0) | 0.0 (0.0-0.0) | 0.0 (0.0-0.0) | 0.0 (0.0-0.0) | 0.0 (0.0-0.0) |
| Enthesitis SPARCC (0-16) | T2 |  |  |  |  |  |  |
| mean (SD) |  | 0.3 (1.2) | 0.3 (1.1) | 0.6 (2.0) | 0.6 (1.9) | 0.4 (1.5) | 0.3 (1.4) |
| median (Q1-Q3) |  | 0.0 (0.0-0.0) | 0.0 (0.0-0.0) | 0.0 (0.0-0.0) | 0.0 (0.0-0.0) | 0.0 (0.0-0.0) | 0.0 (0.0-0.0) |
| Tender joint count (0-68) | T0 |  |  |  |  |  |  |
| mean (SD) |  | 6.2 (7.4) | 6.2 (7.4) | 8.7 (9.0) | 8.7 (9.0) | 6.9 (8.0) | 6.9 (8.0) |
| median (Q1-Q3) |  | 4.0 (1.0-8.0) | 4.0 (1.0-8.0) | 6.0 (2.0-12.0) | 6.0 (2.0-12.0) | 4.0 (2.0-10.0) | 4.0 (2.0-10.0) |
| Tender joint count (0-68) | T1 |  |  |  |  |  |  |
| mean (SD) |  | 2.8 (5.4) | 2.8 (5.6) | 5.5 (8.1) | 5.3 (7.9) | 3.6 (6.5) | 3.5 (6.4) |
| median (Q1-Q3) |  | 1.0 (0.0-3.0) | 1.0 (0.0-3.0) | 2.0 (0.0-8.0) | 2.0 (0.0-7.1) | 1.0 (0.0-4.0) | 1.0 (0.0-4.0) |
| Tender joint count (0-68) | T2 |  |  |  |  |  |  |
| mean (SD) |  | 2.8 (5.3) | 2.7 (5.4) | 4.8 (8.4) | 4.7 (8.2) | 3.4 (6.4) | 3.3 (6.4) |
| median (Q1-Q3) |  | 0.0 (0.0-3.0) | 0.0 (0.0-3.0) | 2.0 (0.0-6.0) | 2.0 (0.0-6.0) | 1.0 (0.0-4.0) | 1.0 (0.0-4.0) |
| Swollen joint count (0-66) | T0 |  |  |  |  |  |  |
| mean (SD) |  | 2.9 (4.0) | 2.9 (4.0) | 3.9 (5.1) | 3.9 (5.2) | 3.2 (4.4) | 3.2 (4.4) |
| median (Q1-Q3) |  | 2.0 (0.0-4.0) | 2.0 (0.0-4.0) | 2.0 (0.0-6.0) | 2.0 (0.0-6.0) | 2.0 (0.0-4.0) | 2.0 (0.0-4.0) |
| Swollen joint count (0-66) | T1 |  |  |  |  |  |  |
| mean (SD) |  | 1.1 (2.8) | 1.1 (2.8) | 1.9 (4.1) | 1.8 (3.9) | 1.3 (3.3) | 1.3 (3.2) |
| median (Q1-Q3) |  | 0.0 (0.0-1.0) | 0.0 (0.0-1.0) | 0.0 (0.0-2.0) | 0.0 (0.0-2.0) | 0.0 (0.0-1.0) | 0.0 (0.0-1.0) |
| Swollen joint count (0-66) | T2 |  |  |  |  |  |  |
| mean (SD) |  | 1.0 (2.8) | 1.0 (2.9) | 1.8 (4.3) | 1.8 (4.2) | 1.3 (3.4) | 1.3 (3.3) |
| median (Q1-Q3) |  | 0.0 (0.0-1.0) | 0.0 (0.0-1.0) | 0.0 (0.0-1.0) | 0.0 (0.0-1.0) | 0.0 (0.0-1.0) | 0.0 (0.0-1.0) |
| ≥3 Comorbidities, n (%) |  | 405 (30) | 450.1 (30) | 229 (41) | 253.9 (41) | 634 (34) | 704.0 (33) |
| DAPSA, mean (SD) |  | 20.3 (12.0) | 20.0 (12.0) | 27.2 (13.9) | 27.0 (14.0) | 22.3 (13.0) | 22.0 (13.0) |
| **Patient-reported characteristics** |  |  |  |  |  |  |  |
| PtGA (NRS 0-10), mean (SD) | T0 | 5.2 (2.3) | 5.2 (2.3) | 7.0 (2.0) | 6.9 (2.0) | 5.7 (2.4) | 5.7 (2.4) |
| PtGA (NRS 0-10), mean (SD) | T1 | 3.9 (2.4) | 3.9 (2.4) | 5.4 (2.6) | 5.4 (2.5) | 4.3 (2.5) | 4.3 (2.5) |
| PtGA (NRS 0-10), mean (SD) | T2 | 3.8 (2.5) | 3.8 (2.5) | 5.3 (2.6) | 5.3 (2.6) | 4.3 (2.6) | 4.2 (2.6) |
| PtGA MSK (NRS 0-10), mean (SD) | T0 | 5.0 (2.3) | 5.0 (2.3) | 6.8 (2.0) | 6.8 (2.0) | 5.6 (2.4) | 5.5 (2.4) |
| PtGA MSK (NRS 0-10), mean (SD) | T1 | 3.8 (2.4) | 3.8 (2.4) | 5.4 (2.5) | 5.3 (2.5) | 4.3 (2.5) | 4.2 (2.5) |
| PtGA MSK (NRS 0-10), mean (SD) | T2 | 3.8 (2.5) | 3.7 (2.5) | 5.3 (2.6) | 5.3 (2.6) | 4.2 (2.6) | 4.2 (2.6) |
| HAQ (0-3), mean (SD) | T0 | 0.8 (0.6) | 0.8 (0.6) | 1.3 (0.6) | 1.3 (0.6) | 0.9 (0.7) | 0.9 (0.7) |
| HAQ (0-3), mean (SD) | T1 | 0.6 (0.6) | 0.6 (0.6) | 1.1 (0.7) | 1.1 (0.7) | 0.8 (0.7) | 0.8 (0.7) |
| HAQ (0-3), mean (SD) | T2 | 0.6 (0.6) | 0.6 (0.6) | 1.1 (0.7) | 1.1 (0.7) | 0.8 (0.7) | 0.8 (0.7) |
| **Treatments** |  |  |  |  |  |  |  |
| B/tsDMARD naïve, n (%) |  | 845 (63) | 959.6 (63) | 300 (54) | 337.4 (54) | 1145 (61) | 1297.0 (61) |
| Inclusion therapy, n (%) |  |  |  |  |  |  |  |
| NSAID |  | 14 (1) | 17.3 (1) | 6 (1) | 6.7 (1) | 20 (1) | 24.0 (1) |
| csDMARD |  | 228 (17) | 251.5 (17) | 70 (13) | 76.5 (12) | 298 (16) | 328.0 (15) |
| bDMARD |  | 987 (74) | 1128.7 (74) | 427 (77) | 478.3 (77) | 1414 (75) | 1607.0 (75) |
| tsDMARD |  | 105 (8) | 121.1 (8) | 51 (9) | 58.9 (9) | 156 (8) | 180.0 (8) |
|  |  |  |  |  |  |  |  |
| b/tsDMARD, biological/targeted synthetic Disease-Modifying Anti-Rheumatic Drug; BMI, Body Mass Index; csDMARDS, conventional synthetic Disease-Modifying Anti-Rheumatic Drugs; DAPSA, Disease Activity in PSoriatic Arthritis; HAQ, Health Assessment Questionnaire; MSK, musculoskeletal; NRS, Numeric Rating Scale; NSAIDS, Non-Steroidal Anti-Inflammatory Drugs; PhGA, Physician Global Assessment; PsA, Psoriatic Arthritis; PtGA, Patient Global Assessment; Q1, first quartile; Q3, third quartile; SD, standard deviation; WHO-5, World Health Organization-5 Well-Being Index. Note: The 'Unknown' category includes 153 women (40 with questionnaire data) and 98 men (18 with questionnaire data). Percentages in Unknown columns are based on available data only. None/mild and moderate/severe depressive symptoms were defined as WHO-5 score ≥29 and <29 (0–100 scale), respectively.  Note: Patients with unknown WHO-5 status excluded from original data for comparison (women: n=153; men: n=98).  Data include variables across baseline and follow-up time points (Baseline [BL], 3 months, 6 months). | | | | | | | |

**Table S3** Multiple Imputation Summary

| **Pre-Imputation Data Characteristics** | | |
| --- | --- | --- |
|  | Total patients, N | 2,139 |
|  | Variables in imputation model | 44 |
|  | Variables with any missing data | 37 |
|  | Auxiliary variables included | 9 |
|  | Complete cases to variables ratio | 54.2:1 (1138 / 21 variables) |
| **Missingness Patterns** | | |
|  | Missingness range across variables | 0-24.1% |
|  | Median missingness | 11.9% |
|  | Baseline variables | 0-13% (median 3.4%) |
|  | 3-month follow-up | 14.4-24.1% (median 18.2%) |
|  | 6-month follow-up | 12.4-22.8% (median 15.4%) |
|  | Average dropout (baseline to 6 months) | 12 percentage points |
| **Analysis-Specific Complete Cases** | | |
|  | MDA at 3 months | 1,251 (58.5%) |
|  | MDA at 6 months | 1,293 (60.4%) |
|  | DAPSA remission at 3 months | 1,138 (53.2%) |
|  | DAPSA remission at 6 months | 1,152 (53.9%) |
|  | Minimum recommended m | 47 (based on 46.8% incomplete) |
| **Missing Data Assumption** | | |
|  | Assumed mechanism | Missing at random (MAR) |
|  | Auxiliary variables to support MAR | Comorbid depression and fibromyalgia (rheumatologist-documented), physician global assessment, dermatology-related quality of life (DLQI), employment status, dactylitis, CASPAR criteria |
|  | Rationale | Missingness predicted by observed disease severity and patient characteristics |
| **Imputation Model Specification** | | |
|  | Software | mice 3.16.0 in R 4.4.2 |
|  | Method | Predictive mean matching (PMM) for all variables |
|  | Number of imputations (m) | 50 |
|  | Iterations per imputation (maxit) | 20 |
|  | Predictor matrix | Default (all variables predict all others) |
|  | Random seed | 12345 |
| **Post-Imputation Quality Indicators** | | |
|  | All imputed datasets complete | Yes |
|  | Convergence achieved | Yes (visual inspection of trace plots) |
|  | Logged warnings | None |
|  | FMI range (all coefficients) | 0.13-0.37 |
|  | FMI for main exposure (WHO-5) | 0.21-0.25 |
|  | Coefficients with FMI > 0.5 | 0 |
| CASPAR, ClASsification criteria for Psoriatic ARthritis; DAPSA, Disease Activity in PSoriatic Arthritis; DLQI, Dermatology Life Quality Index; FMI, Fraction of missing information; m, Number of imputations; MAR, Missing at random; maxit, Maximum iterations; MDA, Minimal Disease Activity; PMM, Predictive Mean Matching; WHO-5, World Health Organization-5 Well-Being Index. Variable-level missingness details are provided in Table S1. Multiple imputation was performed using multivariate imputation by chained equations (MICE) as implemented in the mice R package [1]. PMM was used for all variables to preserve the observed data distribution [2]. The number of imputations (m = 50) was chosen to exceed the percentage of incomplete cases in all analyses, following the recommendation of White et al. [3]. An inclusive modeling strategy was used, incorporating auxiliary variables to make the MAR assumption more plausible and reduce potential bias; this approach is preferred over restrictive strategies that use fewer variables [2,4]. Results were pooled using Rubin's rules [5]. FMI values below 0.5 indicate acceptable imputation performance [3]. The complete cases to variables ratio is calculated as the number of complete cases in the analysis with the highest missingness divided by the number of variables in that analysis; a ratio above 10:1 indicates sufficient information for stable imputation model estimation [2]. References: [1] van Buuren S, Groothuis-Oudshoorn K. mice: Multivariate Imputation by Chained Equations in R. J Stat Softw 2011;45(3):1-67. [2] van Buuren S. Flexible Imputation of Missing Data. 2nd ed. CRC Press; 2018. [3] White IR, Royston P, Wood AM. Multiple imputation using chained equations: Issues and guidance for practice. Stat Med 2011;30(4):377-399. [4] Collins LM, Schafer JL, Kam CM. A comparison of inclusive and restrictive strategies in modern missing data procedures. Psychol Methods 2001;6(4):330-351. [5] Rubin DB. Multiple Imputation for Nonresponse in Surveys. John Wiley & Sons; 1987. | | |

**Table S4** Sensitivity analysis: composite (MDA/DAPSA) vs. objective (SJC/CRP) outcomes

| **Outcome** | **Timepoint** | **Analysis** | **OR (95% CI)** | **p-value** |
| --- | --- | --- | --- | --- |
| MDA (composite) | 3 months | Primary | 1.06 (1.00–1.13) | 0.0514 |
| DAPSA remission (composite) |  | Primary | 1.08 (1.00–1.16) | 0.0403 |
| SJC remission |  | Sensitivity (objective) | 1.00 (0.94–1.07) | 0.9644 |
| CRP normalization |  | Sensitivity (objective) | 0.96 (0.90–1.01) | 0.1438 |
| SJC remission (DAPSA confounder set) |  | Sensitivity (objective) | 1.00 (0.94–1.07) | 0.9216 |
| MDA (composite) | 6 months | Primary | 1.10 (1.03–1.17) | 0.0024 |
| DAPSA remission (composite) |  | Primary | 1.13 (1.06–1.22) | 0.0005 |
| SJC remission |  | Sensitivity (objective) | 0.98 (0.92–1.05) | 0.6162 |
| CRP normalization |  | Sensitivity (objective) | 0.91 (0.86–0.97) | 0.0034 |
| SJC remission (DAPSA confounder set) |  | Sensitivity (objective) | 0.98 (0.92–1.04) | 0.4462 |
| CI, Confidence interval; CRP, C-reactive protein; DAPSA, Disease Activity in PSoriatic Arthritis; MDA, Minimal Disease Activity; OR, Odds ratio; SJC, Swollen joint count. | | | | |

**Figure S1** Flow of psoriatic arthritis patients from the RABBIT-SpA register into the main (multiply imputed) analysis, with the split by observed versus imputed baseline WHO-5.
RABBIT-SpA does not capture data on patients who met eligibility criteria but were not enrolled at participating centers (e.g., due to center-level documentation resource constraints); the number of patients assessed for eligibility is therefore not available. Patients without a baseline physician CRF are not retained in any register dataset and are not represented in this diagram. Withdrawal of consent does not lead to exclusion of already-collected data; affected patients' data are anonymized but retained and used.

The diagram follows the STROBE reporting recommendations for observational studies.

**Figure S2** Proportion of Patients Achieving DAPSA Remission by Category of Depressive Symptoms, Time Point and Sex

**Figure S3** Mean values of the individual components of DAPSA and MDA at baseline, 3 months, and 6 months, stratified by depressive symptom category (Women).

**Figure S4** Mean values of the individual components of DAPSA and MDA at baseline, 3 months, and 6 months, stratified by depressive symptom category (Men).

**Figure S5** DAG: Direct Effect of WHO-5 Well-being Index on Achieving MDA

**Figure S6** DAG: Direct Effect of WHO-5 Well-being Index on Achieving DAPSA Remission

**Abbreviations**

| **Abbreviation** | **Full Form** |
| --- | --- |
| b/tsDMARD | Biological/targeted synthetic Disease-Modifying Anti-Rheumatic Drug |
| BMI | Body Mass Index |
| BSA | Body Surface Area |
| CASPAR | ClASsification criteria for Psoriatic ARthritis |
| CRP | C-reactive protein |
| csDMARDS | Conventional synthetic Disease-Modifying Anti-Rheumatic Drugs |
| DAPSA | Disease Activity in PSoriatic Arthritis |
| DLQI | Dermatology Life Quality Index |
| FMI | Fraction of missing information |
| HAQ | Health Assessment Questionnaire |
| m | Number of imputations |
| MAR | Missing at random |
| maxit | Maximum iterations |
| MICE | Multivariate imputation by chained equations |
| MSK | Musculoskeletal |
| NRS | Numeric Rating Scale |
| NSAIDS | Non-Steroidal Anti-Inflammatory Drugs |
| PhGA | Physician Global Assessment |
| PMM | Predictive Mean Matching |
| PsA | Psoriatic Arthritis |
| PsAID | Psoriatic Arthritis Impact of Disease |
| PsO | Psoriasis |
| PtGA | Patient Global Assessment |
| Q1 | First quartile |
| Q3 | Third quartile |
| SD | Standard deviation |
| WHO-5 | World Health Organization-5 Well-Being Index |
